# Preparation of Polyaminopyridines Using a CuI/l-Proline-Catalyzed C-N Polycoupling Reaction

**DOI:** 10.3390/ma5112176

**Published:** 2012-11-05

**Authors:** Lindomar A. Reis, Carolina B. P. Ligiéro, Acácio A. Andrade, Jason G. Taylor, Paulo C. M. L. Miranda

**Affiliations:** 1 National Institute of Metrology, Quality and Technology, DGTEC-INMETRO, 25250-020 Duque de Caxias-RJ, Brazil; E-Mail: lareis@inmetro.gov.br; 2 Institute of Chemistry, State University of Campinas, UNICAMP, CP 6154, 13083-970 Campinas-SP, Brazil; E-Mail: cligiero@iqm.unicamp.br; 3 Institute of Physics, Federal University of Uberlândia, UFU, 38400-902 Uberlândia-MG, Brazil; E-Mail: aaandrade@pq.cnpq.br; 4 Department of Chemistry, Institute of Biological and Physical Sciences, Federal University of Ouro Preto, UFOP, 35400-000 Ouro Preto-MG, Brazil; E-Mail: jason@iceb.ufop.br

**Keywords:** polyaminopyridines, Ullmann coupling reaction, end-group analysis by XRF

## Abstract

Polyaminopyridines (PAPy) were chemically prepared from amino-bromopyridines by a CuI/l-proline-catalyzed C-N polycondensation reaction. The formation of the polymer was confirmed by GPC, XRD, XRF, FTIR, UV-vis (λ_max_ = 400 nm), ^1^H and ^13^C NMR. The number-average molecular weights (***M_n_***) were estimated by end-group analysis using X-ray fluorescence (up to 6000 Da). TGA analysis of PAPy with higher ***M_n_*** showed greater thermal stability up to 170 ^o^C. Viscosity measurements of polymer in formic acid at 30 ^o^C indicated a polyelectrolyte nature of PAPy solutions. Furthermore, the amorphicity of the material was observed by X-ray diffraction analysis.

## 1. Introduction

Amongst the vast number of conductive polymers described in the scientific literature, polyanilines have attracted great interest due to their excellent thermal and chemical stability which confer high applicability in many devices such as rechargeable batteries [1] and LEDs [2]. Electrochemical and chemical polymerizations of anilines are well-known procedures for synthesizing electroconductive polyaniline and have been studied extensively [3,4,5]. Although several chemical properties of aminopyridine are resembled by aniline, polyaminopyridines (PAPy) have received much less attention than polyanilines for study. In contrast to high yielding polyaniline synthesis [3,4,5], one of the main reasons for the perceived difficulty in the preparation of polyaminopyridines is the fact that chemical yields from their electropolymerizations are often only around 3%–5% [6]. Despite this drawback, applications exploiting the electrical properties of PAPy have already been reported in the literature, as has its use in the construction of batteries [7] and modified electrodes [6].

In the few reports concerning the synthesis of PAPy, the electrochemical route [6,7,8,9] is most used and, frequently, 2-aminopyridine is employed as monomer. Due to the lack of reactivity of the aminopyridine in the oxidative chemical polymerization [10], this method for polymer synthesis has been abandoned. Another pathway towards PAPy synthesis uses the copper-catalyzed Ullmann-type reaction to promote C-N polycoupling between 2,6-diaminopyridines and 2,6-dihalopyridines [11]. Unfortunately, this approach requires high temperatures (~200 °C) and thus restricts the presence of some functional groups that the monomer can bear. In addition, Ullmann-type reactions often require the use of large amounts of copper reagents, which, on scale, leads to problems of waste disposal [12,13]. To overcome these drawbacks of catalysis by copper, several Pd-catalyzed C-N formation methods have been developed [14,15]. In this context, the regio-regulated synthesis of PAPy using Pd-catalyzed amination reaction at 110 °C was reported recently [16]. However, industrial use of methods involving Pd is problematic in many cases due to the air and moisture sensitivity, as well as the high costs of Pd catalysts [17].

In the 1990s, Buchwald and coworkers developed an improved procedure for the Ullmann-type coupling reaction based on the utilization of some bidentate ligands such as 1,10-phenanthroline [18,19]. This modified method made it possible for a catalytic amount of the Copper(I) complex to be used in the presence of cesium carbonate at lower reaction temperatures and thus broadening the applicability of the reaction to a wider variety of functional groups. In past years, other bases were used in combination with different copper sources and ligands in the synthesis of small and large molecules [20,21,22,23]. One of these combinations, CuI/l*-*proline system presented great effectiveness in coupling reactions of bromopyridines with amines [24], which indicated the potential use of this catalytic system for the PAPy preparation. Herein, we report a milder and more economical method for the synthesis of PAPy by CuI/l*-*proline-catalyzed polycoupling reaction of amino-bromopyridines. The reactivities of 5-amino-2-bromopyridine (**1**) and 2-amino-5-bromopyridine (**2**) were evaluated in a systematic study of the reaction conditions. PAPy was chemically characterized and its physical properties were studied.

## 2. Results and Discussion

### 2.1. Solubility and Systematic Study of Polycoupling Reaction

**Scheme 1:**
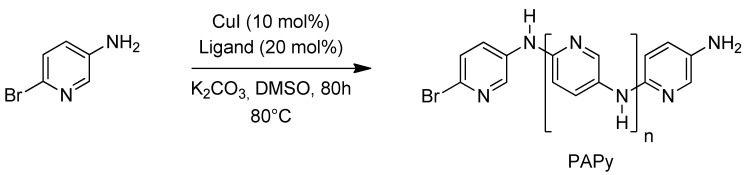
Used conditions for preparing Polyaminopyridines (PAPy) with CuI/l*-proline* system.

Preliminary studies of the polycondensation reaction were performed using amino-bromopyridines as monomer and the best reaction conditions for pyridine systems developed by Ma and coworkers, taken from the arylation protocol of *N*-containing heterocycles [24]. However, the reaction time was increased from 40 to 80 hours, Scheme 1. The dark brown powder obtained as product was similar to that reported by both electrochemical [9] and chemical [16] synthesis of PAPy.

PAPy was insoluble in common organic solvents such as hexane, chloroform, methanol, acetonitrile, *N*-methyl-2-pyrrolidone (NMP), and dimethylformamide (DMF) but very slightly soluble in dimethylsulfoxide (DMSO). The polymer showed reasonable solubility only in strong polar protic solvents such as formic, acetic and trifluoroacetic acids. This behavior is strong evidence of the huge hydrogen bonding network among the polyaminopyridine chains and also for the small extension of branching. Crosslinked chains might also reduce the solubility of the polymer, but in this case, a swelling process was expected to occur even in the case of the organic acids described above. However, in this case, complete solubilization of the polymers took place after 48 hours. The latter two characteristics, low degree of branching and virtually no crosslinking, were indeed expected for Copper(I) cross-coupling reactions [25]. Since this reaction promotes exclusively the substitution of a halogen or a pseudohalogen (Cl, Br, I, and OTf) by a nitrogen atom, any occurrence of crosslinking amongst the polymer chains must be the product of side reaction mechanisms, such as oxidative coupling at high temperature for example, which is an unlikely transformation under these conditions, *i.e.*, nitrogen atmosphere, mild temperature, mild base, and degassed solvents [26,27]. On the other hand, C-H bond activation mediated by Palladium catalysis (Palladium homo-coupling reactions) is an already known reaction and intensively studied nowadays [26,27,28]. In the case of Palladium cross-coupling polymerization reactions, polymer crosslinking may be a real drawback [16].

**Figure 1:**
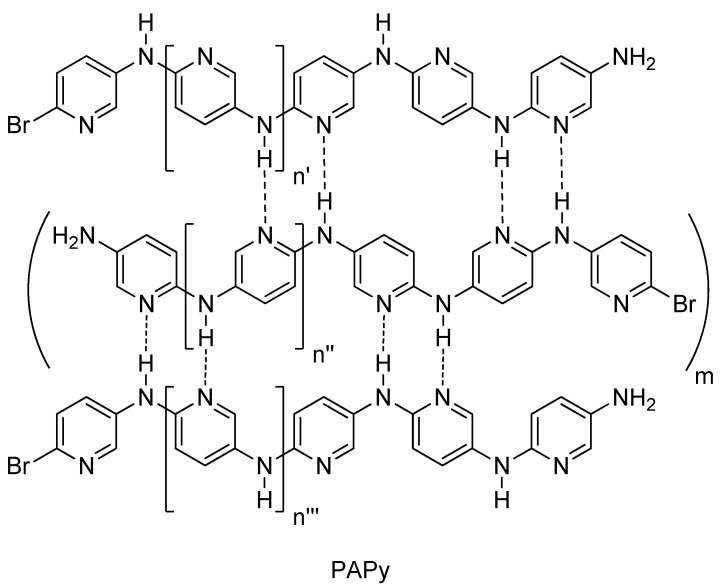
Schematic representation of hydrogen-bonded ladders interchains in PAPy aggregation.

In the case of polyanilines, the aggregation of these polymers in solution is dependent upon the addition of salts such as LiCl and LiBF_4_, which reduce the formation of aggregated chains [29,30]. This behavior was also observed in our case and possibly the formation of hydrogen-bonded ladders and interchains of PAPy [31] (Figure 1) increased the aggregation of polymer chains and therefore decreased the solubility of material in non protic solvents. Conversely, protic solvents such as formic acid and trifluoroacetic acid protonate the nitrogen of the PAPy chains leading to the formation of a positively charged polymer. This in turn decreases the aggregation by electrostatic repulsion and, consequently, increases the solubility of polymer in acidic solution. As will be presented in subsequent sections, viscosity analysis indicated that PAPy in protic solvents display characteristics of a polyelectrolyte solution.

To carry out a systematic optimization of the polycoupling reaction conditions, it was necessary to determine the PAPy molecular weights in order to evaluate the reaction outcome. However, the poor solubility of PAPy in solvents such as NMP, DMF and DMSO made ***M_n_*** determination impracticable for the gel permeation chromatography (GPC) measurements. To increase the polymer solubility, the addition of LiCl to the mixture of organic solvent and polymer was attempted but was not completely successful. Although a visible enhancement of solubility was achieved, the used amount of LiCl was too high and the salt began to crystallize when submitted to GPC conditions. To overcome this drawback, the molecular weights were estimated by end-group analysis with X-ray fluorescence (XRF). The presence of only one Br atom *per* polymeric chain (see Table 1) allowed the estimation of the ***M_n_*** and consequently, the degree of polymerization (***DP***) of the PAPy chains by bromine quantification in the polymer samples.

**Scheme 2:**
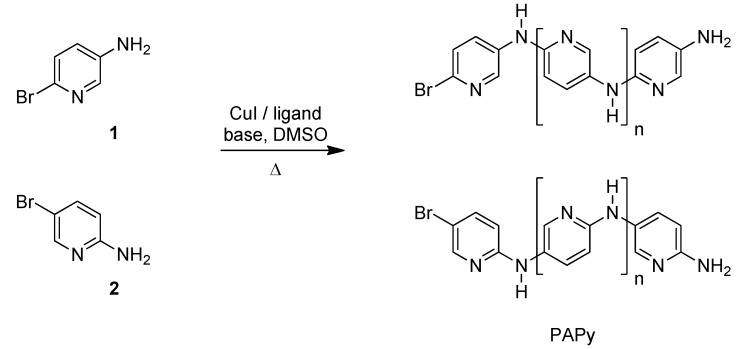
CuI/l*-*proline-catalyzed polycoupling reaction of amino-bromopyridines.

The results of our optimization study are summarized in Scheme 2 and Table 1. Monomer **1** is more reactive than monomer **2** and this led to the formation of PAPy with higher ***M_n_*** and with a more satisfactory yield (comparison among entries 1/2/3 and 12/13/14). The higher reactivity of the amino-bromopyridine (**1**) in comparison to **2** (Scheme 2) was also observed in the synthesis of PAPy by Pd-catalyzed C-N coupling reaction [16]. This behavior was justified by Quantum Chemical calculations as result of partial atomic charges differences in the monomers [16]. In both reactions, the higher partial atomic charge on this carbon atom favors the halide activation step, which is the rate determining step for the copper catalyzed coupling reactions [32]. Lower mass oligomers formed due to the reduced reactivity of the monomer **2** showed good solubility in EDTA aqueous solution used in the washing procedure (entries 12, 13 and 14). Likewise, electrochemically prepared PAPy with low molecular weight (***M_n_*** = 897) presented partial solubility in methanol and water [9].

**Table 1:** Experimental conditions used in PAPy preparation and observed *M_n_*.

Entry	Monomer	Base	Ligand	Condition (^o^C/h)	*DP/M_n_*	Yield (%)
1	1	K_2_CO_3_	l-proline	80/80	21/2013	44
2	1	K_2_CO_3_	l-proline	100/80	46/4313	61
3	1	K_2_CO_3_	l-proline	100/80	64/5969	73^a^
4	1	K_2_CO_3_	l-proline	100/40	32/3025	62
5	1	K_2_CO_3_	none	100/80	7/725	28^b^
6	1	K_2_CO_3_	none	100/80	18/1737	32^b,c^
7	1	K_2_CO_3_	o-phenanthroline	100/80	22/2105	33
8	1	K_2_CO_3_	*N,N*-dimethylglycine	100/80	35/3301	48
9	1	Cs_2_CO_3_	l-proline	100/80	30/2841	45
10	1	K_3_PO_4_	l-proline	100/80	45/4221	80
11	1	K_3_PO_4_	l-proline	100/80	64/5969	87^a^
12	2	K_2_CO_3_	l-proline	80/80	-	0^d^
13	2	K_2_CO_3_	l-proline	80/80	3/357	20^a,e^
14	2	K_2_CO_3_	l-proline	100/80	6/633	23

**^a^** In this case 1.0 g of monomer instead of 0.5 g was employed; ^b^ Reaction without *ligand*; ^c^ DMF was used as solvent; ^d^ The residual solid presented high solubility in the reaction solvent and our standard procedure of washing did not afford material for analysis; ^e^ Material just washed with distilled water.

The importance of using *l-*proline as a chelating agent for catalytic efficiency of the Cu(I) was confirmed by our control reaction performed in the absence of ligand (entries 5 and 6) or using o‑phenanthroline or *N,N*–dimethylglycine as ligands (entries 7 and 8). In the first case, formation of PAPy occurred with lower ***DP*** and worse yields compared with the results obtained in the presence of the l*-*proline (entry 4). Substitution of l*-*proline by o-phenanthroline or *N,N*-dimethylglycine as ligands also furnished lower ***DP*** and yields, but supported the use of XRF in the estimation of the ***M_n_***. The exchange of l*-*proline by o-phenanthroline or *N,N*-dimethylglycine gave more soluble polymers indicating that l*-*proline was not operating as a nucleophile. Although unusual, in some cases nucleophilic ligands may substitute the halogen atom in Copper(I) crosscoupling reactions. The chelating nature of bidentate ligands, such as *l-*proline, is crucial to maintain catalytic activity of the copper-complex and avoid this potential side reaction.

Usually l*-*proline is a better ligand for these reactions giving higher yields and permitting the use of milder reactions conditions [33]. The oligoaminopyridine formation in the reaction without l*-*proline may be an indication that monomer **1** has moderate ability to chelate the copper and promote the crosscoupling reaction. It is known that bidentate molecules with a relatively small bite angle such as the aminopyridines are favored to work in the Cu chelation [33,34].

Amongst inorganic bases used, the results showed that the degree of efficiency for the polymerization reaction increases in the following order K_3_PO_4_ > K_2_CO_3_ > Cs_2_CO_3_ (entries 2, 9 and 10). Although virtually no difference between ***DP*** of the PAPy synthesized using K_3_PO_4_ or K_2_CO_3_ was observed, the phosphate base gave higher yields (entries 2 and 10). Less satisfactory results were obtained from the use of DMF as solvent (comparison between entries 4 and 6). Probably, the lower PAPy solubility in DMF is the responsible factor for observed differences. About 70% of the coupling reaction that lead to the polymer formation occurs within the first 40 hours of reaction (comparison between entries 2 and 4), suggesting that the polymerization proceeds even if the propagating species precipitates out, since these chains are not very soluble in DMSO. Polymer preparation on a larger scale facilitates the process of extraction and purification of PAPy and provides materials with higher ***DP*** (entries 3 and 11). On the basis of these results, the optimal conditions for PAPy synthesis by CuI/l*-*proline-catalyzed C-N polycoupling reaction employ the amino-bromopyridine **1** as monomer, K_3_PO_4_ as the base, DMSO as solvent and carrying out the reaction at 100 °C.

### 2.2. PAPy Characterization

**Figure 2:**
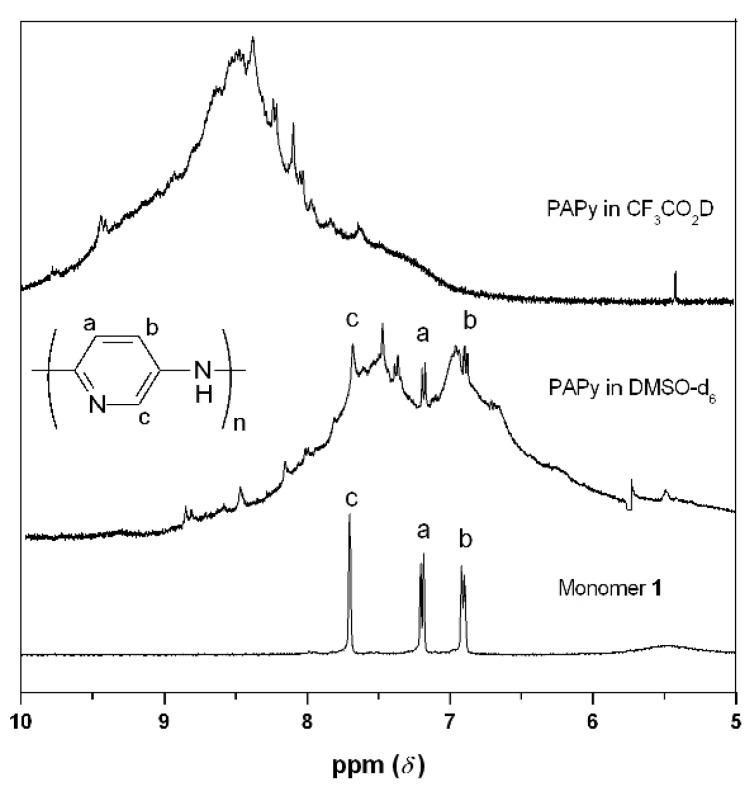
^1^H NMR spectra of the aromatic region of monomer **1** and PAPy (degree of polymerization (***DP***) = 18) at 400 MHz. DMSO-d6 was used as solvent for monomer **1** and PAPy spectra. Deuterated trifluoroacetic acid was also used as solvent for PAPy spectra (upper view).

The higher ***M_n_*** PAPy (***DP*** > 35) were virtually insoluble in DMSO-d6 and it was impossible to acquire ^1^H NMR data. On the other hand, PAPy with lower degree of polymerization (*i.e.*, ***DP*** = 18) were sufficiently soluble in DMSO to confirm polymer formation. The broad signal observed between 6 ppm and 9 ppm is related to aromatic hydrogens of the polymer chain (Figure 2). The displacement of the broad signal for higher chemical shifts in the spectrum obtained in deuterated trifluoroacetic acid indicates the protonation of nitrogen atoms. This increases the electronic repulsion between the chains and explains the solubility of the polymer only in protic acids as previously discussed. The surprisingly broad lines observed in the ^1^H-NMR spectra is another evidence of the intense polymer aggregation, even in the case presented here as illustrated in Figure 2 (***DP*** = 18), suggesting once again the absence of branching in the polymer chains.

**Figure 3:**
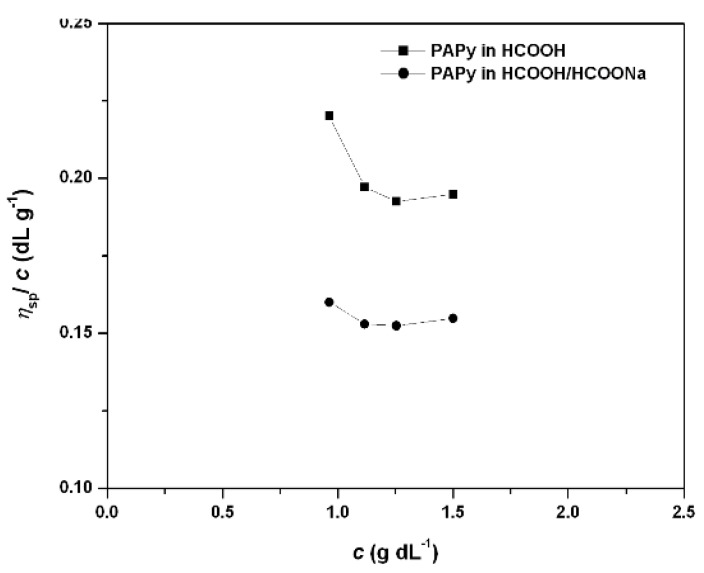
η*_sp_*/*c* versus *c* relationships for PAPy (***DP*** = 64) in HCOOH and in HCOOH/HCOONa (0.1 M) at 30 °C.

Viscosity measurements of PAPy solutions in formic acid showed that the η*_sp_*/*c* versus *c* relationship gives a negative slope, confirming the behavior of polyelectrolyte solution, Figure 3 [29]. Also as expected, an increase in the viscosity of the polymer solutions with molecular weight of the polymer was observed as determined by XRF measurements. The addition of sodium formate promotes counterion binding, leading to decreased repulsive interactions between the ionic moieties along the PAPy chains, which causes a smaller coil size and, consequently, reduction in polymer viscosity as expected for a polyelectrolyte. This phenomenon, known as counterion condensation, effectively reduces the overall charge of the chain and thereby affects its overall conformation [35,36]. Unfortunately, linearization of η*_sp_*/*c* versus *c* plot was not possible in our case, which may be considered an effect of the aggregation enhancement induced by charge lowering in the chain as result of sodium formate addition. Although higher ***M_n_*** PAPy have the highest reduced viscosity values, η*_sp_*/*c* versus *c* plots gave identical negative slopes for all cases examined. This finding is explained by considering that the expansion volume for linear polymers is larger than that for branched polymers, or in other words, linear polymers are able to expand more than branched polymers [35]. So, similar negative slopes would only be possible if all attempted polymerizations gave polymers with the same degree of branching. In our case, given that branching would be an aleatory side reaction, this situation is coherent only with no branching at all, thus, corroborating the preceding observed experimental results.

**Figure 4:**
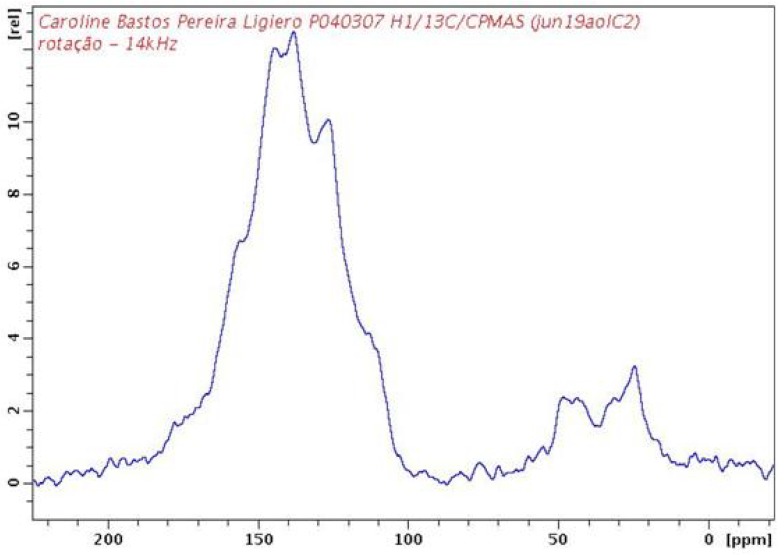
^13^C NMR spectra of PAPy (***DP*** = 18) at 100 MHz.

As shown in the ^13^C NMR spectrum of PAPy, broad signals were observed between 100 ppm and 170 ppm and these can be related to the carbon atoms of the pyridine rings (Figure 4). There is no discrete signal around 170–180 ppm that could be related to the proline carboxylic group. Of course, minimal l*-*proline insertion into the PAPy structure can occur and be underestimated as result of the severe line broadening in both NMR spectra. Such results would imply in the overestimation of calculated ***M_n_*** from the correlation with the bromine content of polymers in these situations. Nevertheless, it is important to note that in the case of polyaniline, even with the use of disaggregating salts, overestimated ***M_n_*** values by almost 30% from GPC measurements can be obtained when compared with the calculated ***M_n_*** observed by light scattering [37]. The structural similarity between polyanilines and polyaminopyridines did not allow the possibility to discard other molecular weight values also overestimated for PAPy by GPC analysis. It is difficult to determine the magnitude of overestimation of calculated ***M_n_*** from the XRF measurements. However, the results obtained by XRF were consistent with the FTIR, UV-vis, X-ray diffraction and thermal analysis as shown below.

**Figure 5:**
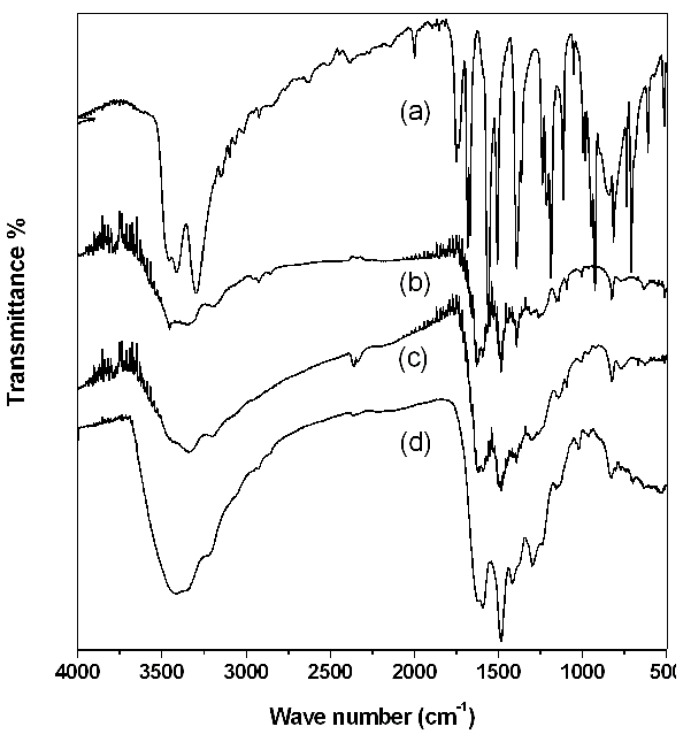
FTIR absorption spectra: (**a**) Monomer **1**; (**b–d**) PAPy: (**b**) ***DP*** = 3; (**c**) ***DP*** = 7; (**d**) ***DP*** = 64.

FTIR spectra for polymers derived from monomer **1** were recorded (Figure 5). The N-H stretching bands and N-H deformation bands of primary amines are seen between 3600 cm^−1^ and 3100 cm^−1^ and 1684 cm^−1^, respectively, in the monomer spectrum; C-Br stretching band has a wave number smaller than 700 cm^−1^ (Figure 5a). Fine structures of PAPy bands (Figure 5b–d) are significantly broader than those observed for monomer **1**, which is consistent with polymer formation. Band broadening in the FTIR spectra have a greater magnitude with higher ***DP*** of the polymer and is in agreement with the end-group analyses by XRF.

**Figure 6:**
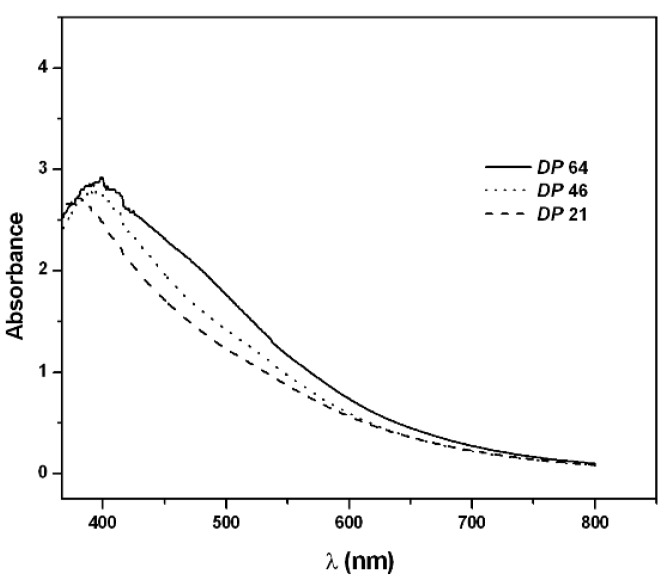
UV-vis spectra of PAPy in trifluoroacetic acid.

PAPy UV-vis spectra show that the absorbance corresponding to π-π* transitions of aromatic rings also increases with higher degree of polymerization estimated by XRF (from 380 nm, ***DP*** = 21, to 400 nm, ***DP*** = 64) which confirms the growth of the PAPy chains, Figure 6. This result is very close to that described by Park and coworkers for PAPy prepared electrochemically (λ_max_ = 375 nm) [6].

**Figure 7:**
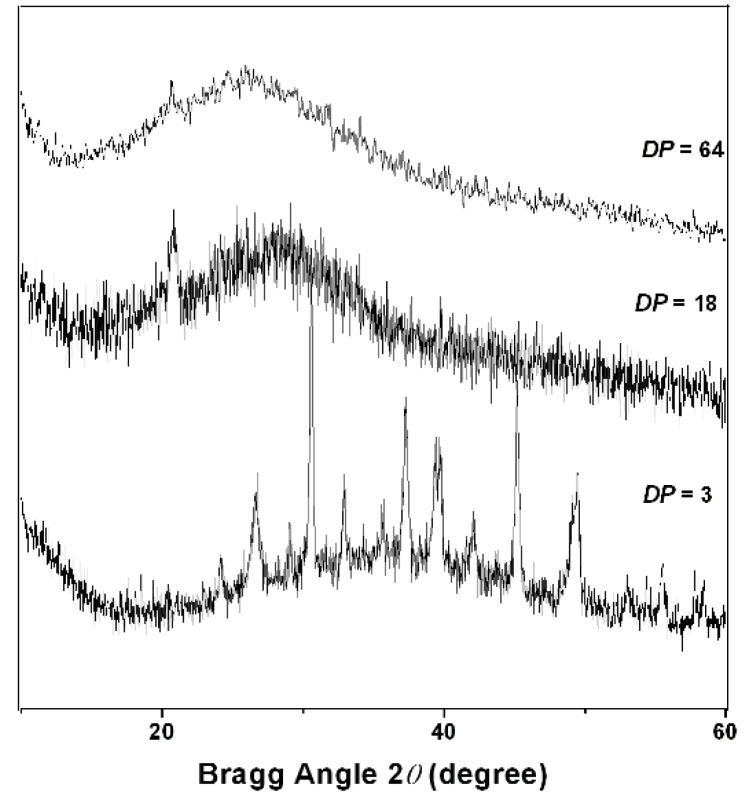
X-ray diffractograms of PAPy with different ***DP***.

X-ray diffractograms of PAPy powder are shown in Figure 7. It was observed that PAPy with higher ***DP*** are predominantly amorphous and diffraction angle tends to be slightly smaller (35° to ***DP*** = 3; 25° to ***DP*** = 64). These results indicate that chain lengthening leads to a disordering of the hydrogen bonding network. Larger chains are able to make not only hydrogen bonds with a greater number of other chains, but also with itself, generating a tangled network.

**Figure 8:**
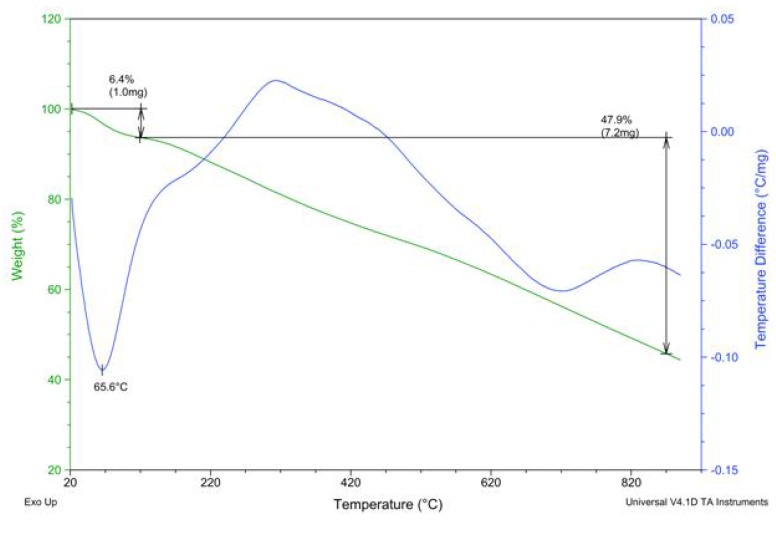

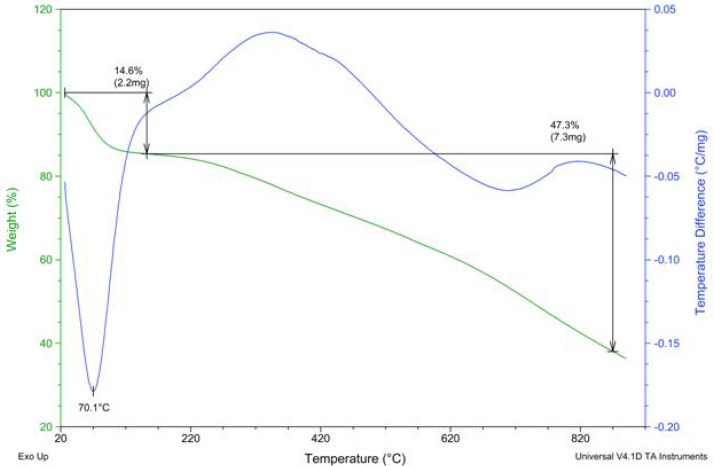
TGA and DTA analysis of the powders of PAPy with ***DP*** = 7 (left panel) and ***DP*** = 64 (right panel) at a heating rate of 10 °C∙min^−1^ in nitrogen.

Thermogravimetric analysis showed that the resistance to thermal degradation presented by PAPy was directly related to the ***DP*** of polymer. PAPy with ***DP*** = 7 began to show an appreciable decomposition at a temperature of about 120 °C, whilst PAPy with ***DP*** = 64 started to decompose at around 170 °C (Figure 8). This small thermal stabilization with chain lengthening also supports the conclusion that larger chains have greater but more disordered hydrogen bonding networks. In summary, all characterization results indicated the formation of the PAPy chains and consistency of the polymer molecular weights as estimated by end-group analysis was established.

## 3. Experimental Section 

All reagents were obtained commercially from Sigma-Aldrich or Acros Organics and used as received. DMSO and DMF were freshly distilled over CaH_2_. CuI must be pre-washed with THF using a Soxhlet extractor before use. 

### 3.1. Measurements

The solubility of the polymers was evaluated using the following method: A polymer powder sample (5 mg) was added into the solvent (0.5 mL) and dispersed thoroughly. After the mixture was stirred continuously for 24 h at room temperature, the solubility of the polymers was characterized semiquantitatively.

IR spectra were obtained with a SHIMADZU 8300 Fourier transform infrared (FTIR) spectrometer on KBr pallets. X-ray diffractograms were recorded with a SEIFERT Model URD-65 diffractometer with monochromatized Co-Kα radiation. The number-average molecular weights (***M_n_***) were estimated through the quantification of bromine content (end-group analysis) in the samples of PAPy by X-ray fluorescence analysis (XRF) using a SHIMADZU EDX-700 spectroscope. Mixtures of KBr/polyaniline were used as standards for construction of the calibration curves for determinations of Br. Polyaniline was prepared by chemical oxidation with (NH)_4_S_2_O_8_ [38]. UV-vis spectra were obtained with a SHIMADZU 1601 PC spectrophotometer, in trifluoroacetic acid solutions. NMR spectra for ^1^H and ^13^C were recorded on a JEOL ECLIPSE + 400 and a Bruker Avance II spectrometers, respectively. All viscosity measurements were carried out in formic acid or formic acid/sodium formate solutions at 30 °C, using a Cannon-Ubbelodhe type viscometer in a thermostabilized water bath.

3.2. Polymerization

Investigation of the PAPy synthesis was carried out through the following standard procedure: A mixture of 5-amino-2-bromopyridine (**1**) or 2-amino-5-bromopyridine (**2**) (500 mg; 3.0 mmol), 0.6 mmol of ligand (l*-*proline, o-phenanthroline, or sarcosine), CuI (55 mg; 0.3 mmol) and an inorganic base (6.0 mmol) in 25 mL DMSO was heated at 80 °C or 100 °C for 80 hours under nitrogen atmosphere. The solvent was removed with high vacuum and the solid residue was collected by filtration, washed with distilled water, aqueous solution of disodic EDTA (pH = 4) and distilled water again, in this order and dried under vacuum to provide a dark brown powder of PAPy.

The amounts of CuI and ligand employed in the reactions were 10 mol% and 20 mol%, respectively, in relation to the monomer stoichiometry. Different bases were tested with a ratio of base to monomer amount 2:1 in all cases.

## 4. Conclusions 

We have developed a CuI/l*-*proline-catalyzed method for the synthesis of PAPy by polycoupling reaction of amino-bromopyridines. This method is attractive due to the use of inexpensive reagents and mild conditions. Our results suggest that aggregate formation between the PAPy chains by hydrogen bonding is one of the responsible factors for the low polymer solubility in common organic solvents. Calculated ***M_n_*** values by end-group analysis by XRF were in agreement with the results obtained from chemical and physical analysis of polymers. Overall, the use of copper/ligand catalysts is very promising for the synthesis of amino-aromatic polymers because of the wide variety of copper sources and ligands available which increases the scope for possible monomers that can be used in polycoupling reactions.
